# The novel interplay between CD44 standard isoform and the caspase-1/IL1B pathway to induce hepatocellular carcinoma progression

**DOI:** 10.1038/s41419-020-03158-6

**Published:** 2020-11-09

**Authors:** Jun Li, Yongwei Zhang, Ran Ruan, Wei He, Yeben Qian

**Affiliations:** 1 Department of General Surgery, First Affiliated Hospital of Anhui Medical University, Hefei, 230022 China; 2 Department of Immunology, School of Basic Medical Sciences, Anhui Medical University, Hefei, 230032 China

**Keywords:** Cancer genetics, DNA metabolism

## Abstract

Accumulating data indicate caspase-1 (CASP1), one of the inflammatory caspases, promotes hepatocellular carcinoma (HCC) progression in tumor proliferation, invasion, EMT phenotype and sorafenib resistance. However, the molecular basis of regulating caspase-1 expression and caspase-1/IL1B (interleukin-1β) pathway in HCC remains unclear. Here, we demonstrated the novel interplay between caspase-1/IL1B activation and cluster differentiation 44 standard isoform (CD44s) in HCC. In this study, we observed that CD44s is responsible for caspase-1/IL1B activation both in HCC tissues and five HCC cell lines. In normoxia conditions, CD44s knockdown repressed the activation of caspase-1/IL1B via stimulating AMPK-mediated autophagy. Moreover, our data suggested that p62-induced autophagic degradation of caspase-1 accounted for caspase-1/IL1B inactivation in CD44s deficient cells. Administration of recombinant human IL1B could rescue impaired proliferation, invasion, and EMT phenotype in CD44s deficient HCC cells. Lastly, hypoxia-mediated caspase-1/IL1B overexpression could be abolished by CD44s downregulation through decreasing HIF1A and enhancing autophagic activity. Overall, targeting CD44s is a novel inhibitory mechanism of caspase-1/IL1B expression, both in normoxia and hypoxia conditions.

## Introduction

Hepatocellular carcinoma (HCC) is an extremely malignant tumor with a 5-year survival rate of only around 18%^1^. The high rate of intrahepatic metastasis and postsurgical recurrence is the leading cause of poor prognosis of patients with HCC. Therefore, it is imperative to explore the molecular basis of HCC progression. Inflammatory caspase-1 has been demonstrated to be activated in immune responses and responsible for the production of inflammatory cytokines, such as IL1B (interleukin-1β) and IL18 (interleukin-18)^2^. Emerging evidence indicates that caspase-1/IL1B activation exerts crucial roles in the development of various tumors^3–6^. Allan Tsung et al. confirmed that caspase-1 activation is essential for hypoxia-induced invasion and metastasis of HCC^5^. Moreover, caspase-1 has been reported to be associated with HCC inflammatory microenvironment, epithelial–mesenchymal transition (EMT) phenotype, and sorafenib treatment, which reduces caspase-1/IL1B expression by suppressing TLR4/stat3/SUMO1 signaling pathway^7^. However, the regulatory mechanisms of caspase-1/IL1B activation in HCC are not yet fully elucidated.

Cluster differentiation 44 (CD44), a transmembrane receptor for hyaluronic acid (HA), is one of the most common cancer stem cells (CSCs) marker^8^. CD44 is encoded by a highly conserved gene, and its pre-messenger RNA could be alternatively spliced into mature mRNAs encoding variant isoforms (CD44v) or standard isoform (CD44s). CD44s have been reported to be tightly related to the poor prognosis of patients with HCC and contribute to tumor metastasis and chemo-resistance^9–12^. Moreover, Bousoik et al.^13^ showed that shedding extracellular domain of CD44 reduced caspase-1/IL1B activation in macrophage, which suggesting CD44s might be an upstream regulator of caspase-1/IL1B. However, the underlying molecular basis of the interplay between CD44s and caspase-1/IL1B remains unclear.

Autophagy is a conserved catabolic pathway by fusing proteins, damaged macromolecules, and organelles with lysosomes in order to maintain energy homeostasis^14–16^. Autophagy-mediated selective degradation of miRNAs or proteins has been demonstrated to be crucial mechanisms in controlling HCC proliferation and metastasis^17–19^. A previous study indicated that caspase-1 could be interacted with p62, the core effector of the autophagic degradation system^20^. Therefore, autophagy might be a potential molecular mechanism between CD44 and caspase-1/IL1B.

The aim of this study is to explore the underlying mechanism of caspase-1/IL1B activation in HCC and further determine whether CD44s modulates caspase-1/IL1B activation through the autophagic degradation of caspase-1. Our findings suggest that CD44s knockdown could suppress caspase-1/IL1B by enhancing autophagic degradation of caspase-1, which results in HCC growth inhibition in normoxia conditions. Moreover, CD44s deficiency abrogates the hypoxia-induced activation of caspase-1/IL1B. Overall, the novel regulatory mechanism of caspase-1/IL1B and CD44s, both in normoxia and hypoxia conditions, will deepen our understanding of HCC progression and provide new targets for pharmacotherapeutic intervention.

## Materials and methods

### Patients and specimens

Tumor samples were achieved from 50 patients who had undergone curative resection between 2017 and 2019 and were pathologically confirmed HCC at the first affiliated hospital of Anhui Medical University. The clinical signatures of 40 patients are summarized in Table 1. Informed consent was obtained from each recruited patient, and the protocol was approved by the Institutional Research Ethics Committee.

**Table 1:** The clinicopathologic characteristics of 40 cases of HCC patients.

	CD44s			Caspase-1		
	Low	High	*P* value	Low	High	*P* value
*n* (%)	22 (55.0)	18 (45.0)		20 (50.0)	20 (50.0)	
*Gender*						
Male	17 (42.5)	11 (27.5)	0.267	15 (37.5)	13 (32.5)	0.490
Female	5 (12.5)	7 (17.5)		5 (12.5)	7 (17.5)	
*Age, y*						
≤50	16 (40.0)	13 (32.5)	0.972	15 (37.5)	14 (35.0)	0.723
>50	6 (15.0)	5 (12.5)		5 (12.5)	6 (15.0)	
*HBsAg*						
Negative	9 (22.5)	8 (20.0)	0.822	9 (22.5)	8 (20.0)	0.749
Positive	13 (32.5)	10 (25.0)		11 (27.5)	12 (30.0)	
*AFP, ng/ml*						
≤20	14 (35.0)	7 (17.5)	0.119	13 (32.5)	8 (20.0)	0.113
>20	8 (20.0)	11 (27.5)		7 (17.5)	12 (30.0)	
*Cirrhosis*						
No	18 (45.0)	8 (20.0)	0.014	14 (35.0)	12 (30.0)	0.507
Yes	4 (10.0)	10 (25.0)		6 (15.0)	8 (20.0)	
*Tumor size, cm*						
≤5	11 (27.5)	8 (20.0)	0.726	12 (30.0)	8 (20.0)	0.206
>5	11 (27.5)	10 (25.0)		8 (20.0)	12 (30.0)	
*Tumor number*						
Single	15 (37.5)	11 (27.5)	0.641	12 (30.0)	14 (35.0)	0.507
Multiple	7 (17.5)	7 (17.5)		8 (20.0)	6 (15.0)	
*Vascular invasion*						
No	18 (45.0)	9 (22.5)	0.033	17 (42.5)	10 (25.0)	0.018
Yes	4 (10.0)	9 (22.5)		3 (7.5)	10 (25.0)	
*TNM stage*						
I–II	17 (42.5)	7 (17.5)	0.014	16 (40.0)	8 (20.0)	0.010
III–IV	5 (17.5)	11 (27.5)		4 (10.0)	12 (30.0)	

Abbreviations: *AFP* alpha fetoprotein, *HBsAg* hepatitis B surface antigen.

### Animals and reagents

Male BALB/c nu/nu mice (6–8 weeks old) were purchased from Shanghai Institute of Material Medicine, Chinese Academy of Science. All procedures of animals adhered to guidelines provided by the Animal Ethics Committee of the first affiliated hospital of Anhui Medical University. Nude mice were randomly divided into four groups. Antibodies and reagents were listed in Supplementary Table 1.

### Cell culture

The human HCC cell line HCCLM3, Huh7, Bel7402, MHCC-97H, and SMMC-7721 were all purchased from the Cell Bank of the Chinese Academy of Sciences (Shanghai, China) and routinely authenticated by STR profiling. Cells were cultured in Dulbecco’s Modified Eagle Medium (DMEM) supplemented with 10% fetal bovine serum (Gbico, USA), 100 U/ml penicillin and 100 μg/ml streptomycin.

### Orthotopic implantation model in vivo

In total, 5 × 10^6^ Huh7-shCon or Huh7-shCD44 cells suspended in 200 μl PBS were injected subcutaneously into mice. After 14 days, two group mice were sacrificed, and tumor tissues were minced into small pieces of equal volume (2 × 2 × 2 mm^3^) and transplanted into the livers of 18 different mice (6 in all 3 groups). All mice were monitored every 3 days and killed 5 weeks later. Lungs and livers were removed, pictured, and embedded in paraffin, and the total number of lung metastases was counted under the microscope.

### Immunoblot analysis

Briefly, total protein was isolated with radioimmunoprecipitation assay buffer, separated by sodium dodecyl sulphate-polyacrylamide gel electrophoresis, and transferred onto polyvinylidene fluoride membranes. Blocked with 5% bovine serum albumin in tris-buffered saline, the membrane was incubated with primary antibodies at 4 °C overnight and then with horseradish peroxidase (HRP)-conjugated secondary antibodies. Signals were imaged by Tanon System.

### Quantitative polymerase chain reaction

Briefly, total RNA of HCC tissues and cells were isolated by Trizol reagent (Life Technology), and 2 μg of total RNA was reverse-transcribed (Takara). Quantitative polymerase chain reaction (Q-PCR) experiments were performed in triplicate by using SYBR Green real-time PCR master mix (Takara) on the Real-Time PCR system (ABI ViiATM7, USA). Q-PCR primers are listed in Supplementary Table 2.

### shRNA and siRNA transfection

For stably knockdown of CD44 with lentivirus shRNA, 2 × 10^5^ cells were plated onto 6-well plates. After 24 h, the liquid containing shRNA was added to the cultural medium according to protocol. AMPK siRNA, CD44 siRNA, and control siRNA (Riobio, China) were transfected into cells using lipofectin 2000 according to the manufacturer’s instructions. Followed by siRNA or mimic treatment for 48–72 h, total protein and RNA were extracted for western blot and Q-PCR analysis.

### Immunofluorescence

Immunofluorescence was performed as previously described^21^. Briefly, cells were seeded in 24-well dishes and fixed by 4% paraformaldehyde and stained with primary antibodies followed by FITC-conjugated secondary antibodies. Images were collected by fluorescent microscopy (Leica, Germany) and processed with ImagePro Plus.

### Immunohistochemistry

IHC of HCC samples was conducted as previously described^7^. Briefly, after incubated with primary antibodies and HRP-conjugated secondary antibodies, the sections were stained by an Envision System (DakoCytomation).

### Caspase-1 activity assay

Caspase-1 activity assays (Beyotime, China) were performed according to manufacturer’s instructions.

### IL1B ELISA

IL1B concentration was measured in the culture medium according to the paper instruction by using ELISA Assay (MultiSciences, China).

### Cell proliferation

Cell proliferation was evaluated by Edu assays (KeyGene, China). Cells transfected with CD44 siRNA or control siRNA were seeded in 96-well plates at 5 × 10^3^ per well and cultured 24 h. For Edu assays, all performances were according to the manufacturer’s instructions.

### Invasion assays

The invasive ability of HCC cells was measured via 24-well transwell chambers separated by polycarbonate membranes with 8 µm pores and precoated with Matrigel (Corning, USA). The lower chamber was filled with complete DMEM as a chemoattractant. Cells transfected with CD44 siRNA or control siRNA in serum-free medium were seeded at 5 × 10^4^ in the upper chamber and incubated at 37 °C in a humidified incubator containing 5% CO_2_. Cells that migrated to the underside of the membrane were fixed and stained with Giemsa (Sigma-Aldrich, USA), detected, and calculated with a microscope (Leica, Germany).

### Co-immunoprecipitate

Immunohistochemistry (IHC) of HCC samples was conducted as previously described^7^. Huh7 cells transfected with CD44 siRNA or control siRNA for 48 h were harvested and lysed in ice-cold IP buffer (KeyGene, China) for 30 min. Total protein extracts were centrifuged at 12,000*g* for 10 min at 4 °C. A 500 μl of the total lysate was incubated at 4 °C with 2 μg of corresponding antibodies or IgG as control and 50 μl protein A/G beads (Santa Cruz, USA) to immunoprecipitate caspase-1, p62, and LC3B. The interacted complexes were then washed three times with lysis buffer. After centrifuged at 12,000*g* for 10 min at 4 °C, pellets were suspended in 100 μl lysis buffer and boiled with 1× sodium dodecyl sulfate loading buffer and then processed by immunoblot analysis.

### Transmission electron microscopy

Huh7 cells seeded onto a six-well plate were fixed with a fixative buffer containing 2% paraformaldehyde and 2.5% glutaraldehyde in 0.1 M PBS. After embedded, samples were cut into 0.12 μm sections and stained with 0.2% lead citrate and 1% uranyl acetate. The images were detected by a JEOL TEM-2000 EXII (JEOL, Japan).

### Statistical analysis

Fisher’s exact tests and *χ*2 tests were used to evaluate correlations between clinical-pathological characters and expressions of caspase-1 or CD44s. The Student’s *t* test was used to make a statistical comparison between variables. GraphPad Prism 6.0 was used for statistical analyses. *P* value < 0.05 was defined as statistically significant. All analyses were carried out on normally distributed data.

## Results

### CD44s positively correlates with caspase-1

**Fig. 1:**
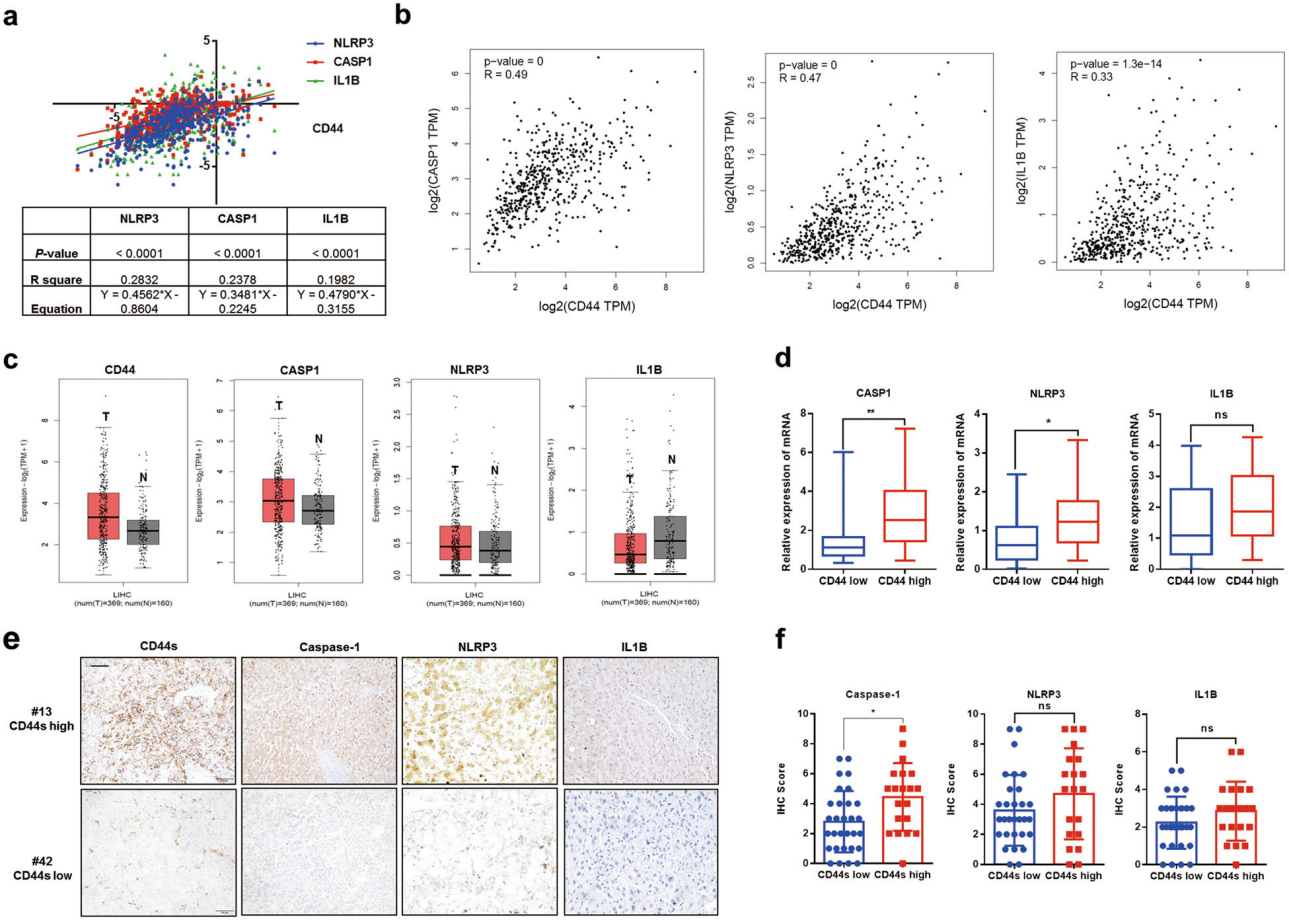
CD44s is positively related to caspase-1/IL1B pathway. **a** Positive correlation between CD44 mRNA and genes involved in the caspase-1/IL1B pathway, such as CASP1, NLRP3, and IL1B. Relevant data were extracted from UCSC Xena for correlation analysis. **b** Positive correlation between CD44 mRNA and genes involved in the caspase-1/IL1B pathway, such as CASP1, NLRP3, and IL1B. Relevant data were extracted from GEPIA for correlation analysis. **c** Expression of CD44 mRNA and genes involved in the caspase-1/IL1B pathway in tumor or non-tumor tissues. Relevant data were extracted from GEPIA. “T” represents tumor tissues referred to red column. **d** mRNA expression of genes involved in the caspase-1/IL1B pathway in HCC tissues with high or low CD44 mRNA expression. *n* = 50. **e**, **f** Representative images of IHC staining CD44s, NLRP3, caspase-1, and IL1B in HCC samples and IHC scores were analyzed. Scale bars, 100 µm. Data are means ± SEM from 3 independent experiments, * means *p* < 0.05, ** means *p* < 0.01, *** means *p* < 0.001 by unpaired student *t* test.

Accumulating data demonstrate the critical role of NLRP3 in modulating caspase-1/IL1B pathway^6,22,23^. To determine the mRNA relationship between CD44 and genes involved in the caspase-1/IL1B pathway, including CASP1, NLRP3, and IL1B, we firstly analyzed mRNA data collected from UCSC Xena and GEPIA, results of which indicated that CD44 was tightly associated with genes involved in the caspase-1/IL1B pathway (Fig. 1a, b). According to data from GEPIA, CD44 and CASP1 were both overexpressed in HCC tissues compared to non-HCC tissues, suggesting a closer association between CD44 and CASP1 rather than NLRP3 or IL1B at mRNA level (Fig. 1c). To further verify the relationship between CD44 and caspase-1/IL1B, Q-PCR analysis was performed in 50 HCC tissues, and we confirmed that CD44 was positively associated with CASP1 and NLRP3 (Fig. 1d). Through IHC experiments, we found that CD44s, NLRP3, caspase-1, and IL1B were expressed in most tumor cells, and we demonstrated the positive relationship between CD44s and caspase-1 by quantifying IHC results (Fig. 1d–f, Fig. S1a). Taken together, our findings suggested that there was a positive correlation between CD44s and caspase-1 in HCC specimens.

### Downregulation of CD44s suppresses the caspase-1/IL1B pathway in vitro

**Fig. 2:**
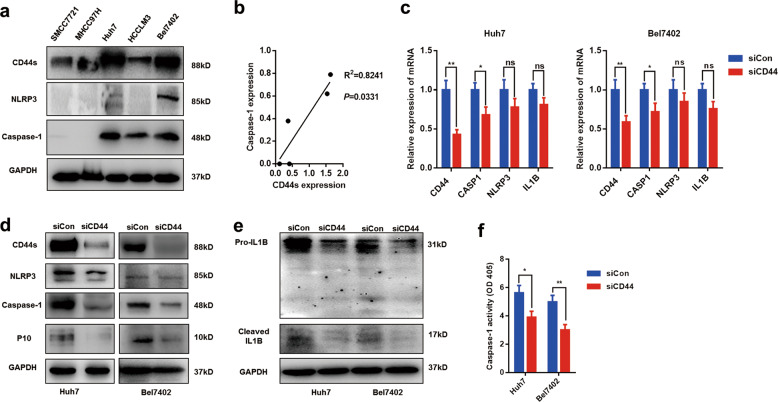
Targeting CD44s inhibits the caspase-1/IL1B pathway. **a**, **b** Protein level of CD44s, caspase-1, and NLRP3 in five HCC cell lines were detected by western blot. The ratio of CD44s/GAPDH and caspase-1/CD44s was analyzed for the correlation. **c** Q-PCR analysis was performed to measure the mRNA expression of CASP1, NLRP3, and IL1B in control (siCon group) or CD44s deficient cells (siCD44 group). **d**, **e** Immunoblot analysis indicated that the caspase-1/IL1B pathway was inactivated in siCD44 cells. P10 refers to the active form of caspase-1. Cleaved IL1B refers to the mature form of IL1B. **f** Caspase-1 activity analysis showed CD44s knockdown inactivated caspase-1 capacity. Data are means ± SEM from 3 independent experiments, * means *p* < 0.05, ** means *p* < 0.01, *** means *p* < 0.001 by unpaired student *t* test.

Furthermore, we explored the relationship between CD44s and caspase-1/IL1B in vitro. Protein levels of CD44, NRLP3, and caspase-1 were tested and quantified in five HCC cell lines (Fig. 2a, b). We found CD44s expression was positive with caspase-1 expression, which was in accordance with the findings from HCC tissues (Fig. 2b). To determine the role of CD44s in modulating caspase-1/IL1B signaling, two highly expressing caspase-1 HCC cell lines, Bel7402, and Huh7, were treated with specific CD44 siRNA to silence the expression of CD44s. mRNA expression of caspase-1 rather that NLRP3 or IL1B was significantly downregulated in both CD44s deficient cell lines, which revealed that caspase-1 was regulated by CD44s (Fig. 2c). Moreover, through immunoblot analysis, we observed that the active form of caspase-1 (p10) and cleaved IL1B were dramatically reduced in two siCD44 cell lines, suggesting that CD44s knockdown led to inactivation of the caspase-1/IL1B pathway (Fig. 2d, e). To further confirm the role of CD44s in caspase-1 expression, a colorimetric assay was performed, and we found that caspase-1 activity was significantly downregulated in CD44s deficient cells (Fig. 2f). Collectively, these observations showed that CD44s was indispensable for caspase-1 activity and targeting CD44s inactivated caspase-1/IL1B pathway by repressing caspase-1 expression.

### Autophagy is responsible for caspase-1/IL1B inhibition in CD44s deficient cells

**Fig. 3:**
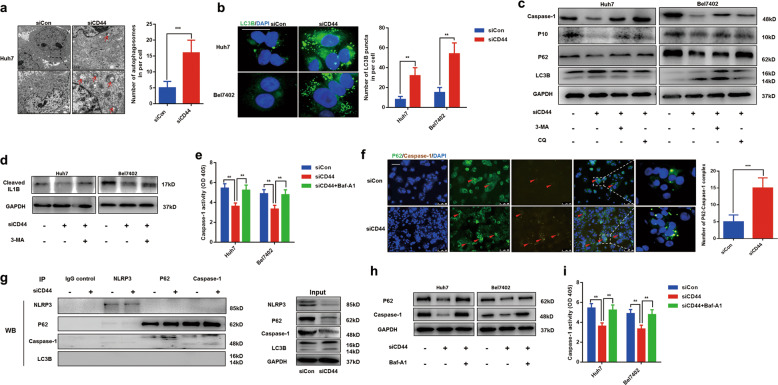
Autophagy induced by targeting CD44s accounts for the caspase-1/IL1B suppression. **a** Autophagesomes obtained by transmission electron microscopy in control (siCon) or CD44s deficient cells (siCD44) were represented, and images were analyzed. **b** Immunofluorescence staining LC3B was performed in two groups, and numbers of LC3B puncta were calculated for analysis. **c**, **d** Immunoblot analysis was performed, and results indicated that downregulation of CD44s contributes to autophagy induction, and autophagy inhibitors such as 3-MA or CQ rescued the impaired caspase-1/IL1B pathway in siCD44 cells. CD44s deficient cells (siCD44) were treated with 3-MA (10 mM) or CQ (50 μM) for 24 h. **e** Caspase-1 activity analysis showed 3-MA treatment rescued damaged caspase-1 capacity in siCD44 cells. **f** Representative images of the distribution of p62 and caspase-1, both determined by immunofluorescence. Numbers of p62–caspase-1 complex were calculated for analysis. Scale bars, 100 µm. **g** Co-IP analysis of caspase-1, NLRP3, LC3B, and p62 from cell lysates immunoprecipitated by NLRP3, caspase-1, LC3B, p62, or lgG antibody, respectively. lgG antibody was indicated as control. **h** Immunoblot analysis was performed, and results indicated that Baf-A1 treatment inhibits caspase-1 decrease in CD44s deficiency cells. CD44s deficient cells (siCD44) were treated with Baf-A1 (100 nM) for 24 h. **i** Caspase-1 activity analysis showed Baf-A1 treatment rescued damaged caspase-1 capacity in siCD44 cells. Data are means ± SEM from 3 independent experiments, * means *p* < 0.05, ** means *p* < 0.01, *** means *p* < 0.001 by unpaired student *t* test.

Next, we seek to investigate the molecular basis of CD44s in regulating the caspase-1/IL1B pathway. Autophagy is a conserved catabolic process and visualized as double membranes^14,15^. Interestingly, we found that the number of double-membrane vesicles containing cytoplasmic components in CD44s deficient cells was increased compared to normal cells by transmission electron microscopy, suggesting knockdown of CD44s would contribute to autophagy induction (Fig. 3a). Immunofluorescence staining LC3B was performed, and results showed the number of LC3B puncta was upregulated in two siCD44 cell lines (Fig. 3b). Moreover, results from Q-PCR and immunoblot analysis further confirmed that targeting CD44s resulted in autophagy induction (Fig. S2a, b). To demonstrate the role of autophagy in regulating the caspase-1/IL1B pathway, Bel7402 siCD44, and Huh7 siCD44 cells were pretreated with autophagy inhibitor 3-Methyladenine (3-MA) or chloroquine (CQ) for 24 h. With the decrease of autophagy measured by the ratio of LC3II/LC3I, impaired expressions of caspase-1, p10, and cleaved IL1B in CD44s deficient cells were all partly rescued, which suggested autophagy was essential for caspase-1/IL1B repression in CD44s deficient cells (Fig. 3c, d). Furthermore, 3-MA treatment recovered damaged caspase-1 activity in two siCD44 cell lines, which confirmed that autophagy accounted for the inactivation of the caspase-1/IL1B signaling pathway (Fig. 3e). All the above results demonstrated that CD44s deficiency-induced autophagy was responsible for the decrease of the caspase-1/IL1B pathway.

Mounting evidence has demonstrated the critical of autophagy in tumor progression through degrading specific proteins or miRNAs. Ubiquitinated proteins can be recognized by p62 during the autophagic degradation process^21^. The complex of specific proteins and p62 will be recruited to autophagosomes and then fused with lysosomes for degradation. A previous study has confirmed that caspase-1 could interact with p62 and the interaction promotes caspase-1-mediated cleavage of p62 in macrophage, which leads to inflammation resolution^20^. We speculated that p62-mediated degradation of caspase-1 might be responsible for CD44s deficiency-mediated suppression of the caspase-1/IL1B pathway. To test this hypothesis, immunofluorescent experiments staining p62 and caspase-1 were performed, and results indicated that the number of p62–caspase-1 complex was increased in CD44s deficient cells (Fig. 3f). Moreover, to confirm the increase of co-localization of caspase-1 and p62, co-immunoprecipitation (Co-IP) experiments were carried out and results demonstrated that caspase-1 instead of NLRP3 or LC3B, was interacted to p62 and targeting CD44s significantly promoted the complex formation of p62 and caspase-1 (Fig. 3g). In order to repress autophagic degradation of caspase-1, Bafilomycin A1 (Baf-A1), an inhibitor for the maturation of autolysosomes, was used, and we found Baf-A1 treatment significantly overexpressed caspase-1 and restored caspase-1 activity in CD44s deficient cells (Fig. 3h, i). To summarize, we confirmed that p62 mediates selective autophagic degradation of caspase-1 is responsible for the decline of the caspase-1/IL1B pathway during autophagy induction caused by CD44s deficiency.

### AMPK/mTOR signaling pathway is essential for CD44s deficiency-mediated autophagy induction

**Fig. 4:**
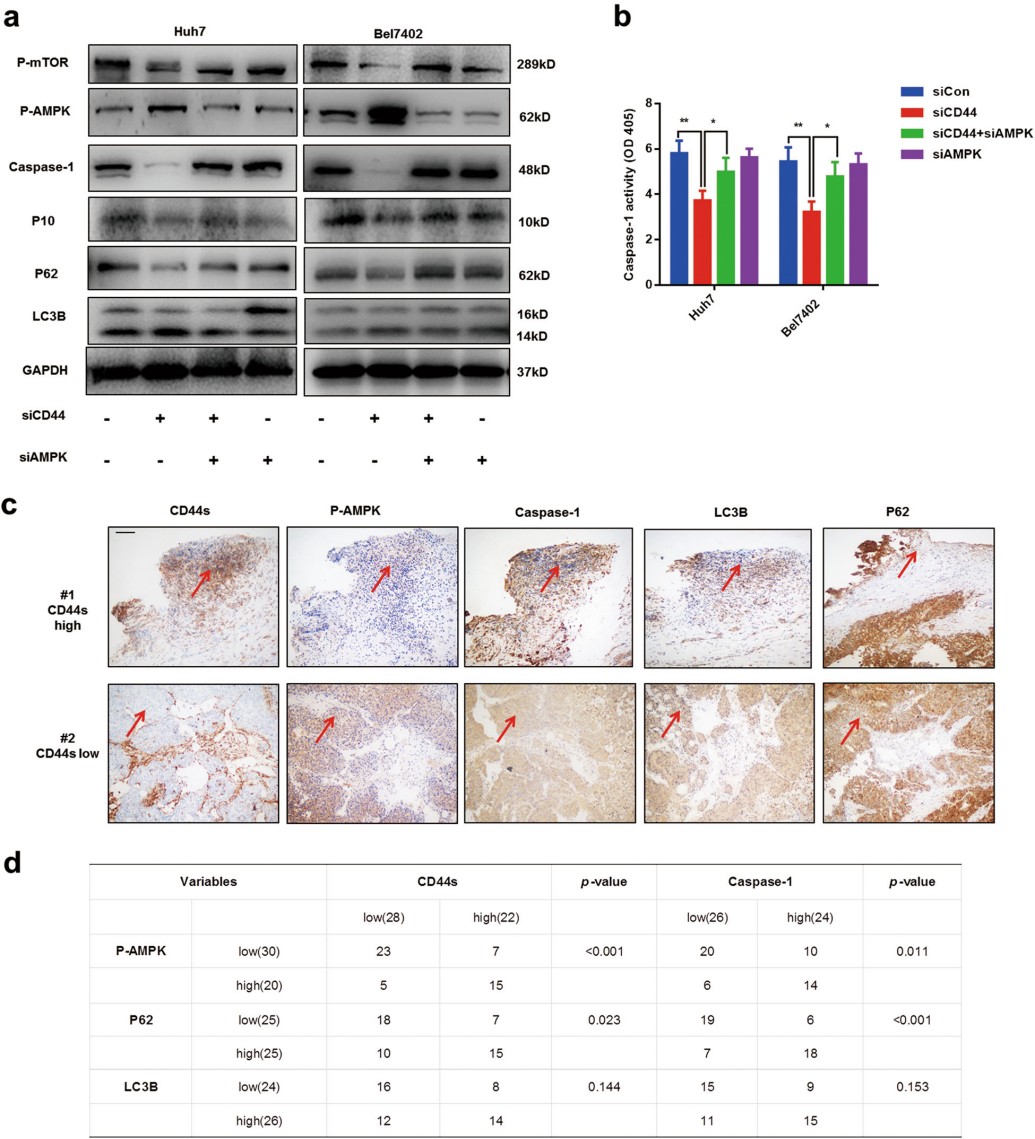
Activation of AMPK/mTOR signaling is essential for autophagy induction and caspase-1 repression in CD44s deficient cells. **a** Immunoblot analysis revealed that AMPK/mTOR signaling activated by CD44s knockdown was responsible for autophagy induction and caspase-1 repression. **b** Caspase-1 activity analyses were performed in four groups and results suggested that AMPK siRNA treatment rescued impaired capase-1 capacity through suppressing autophagy in siCD44 cells. **c** Representative images of IHC staining CD44s, caspase-1, autophagy markers (LC3B and P62), and p-AMPK in of HCC samples, and the same sections were marked by red arrows which referred to high or low expression, correspondingly. **d** IHC results were quantified and analyzed. Scale bars, 100 µm. Data are means ± SEM from 3 independent experiments, * means *p* < 0.05, ** means *p* < 0.01, *** means *p* < 0.001 by unpaired student *t* test.

Many signaling pathways have been proven to modulate autophagy in various conditions, including PI3K/AKT/mTOR and AMPK/mTOR^24–26^. However, the underlying molecular mechanism between CD44s and autophagy has been rarely reported. Recently, CD44 has been demonstrated in regulating energy metabolism via stimulating SRC/AKT/AMPK signaling^27^. Therefore, we thought AMPK/mTOR might be the main cause of autophagy induction in CD44s deficient cells. Results from immunoblot analysis showed that the AMPK/mTOR signaling pathway was activated in CD44s deficient cells (Fig. 4a). To demonstrate the role of AMPK/mTOR pathway in autophagy induction and caspase-1/IL1B suppression, specific AMPK siRNA were designed and used to inhibit AMPK expression. Following the treatment with AMPK siRNA, autophagic capacities in CD44s deficient cells were decreased, and impaired caspase-1/IL1B pathway was recapitulated correspondingly (Fig. 4a). Furthermore, the colorimetric caspase-1 assay confirmed the recovery of caspase-1 activity by AMPK siRNA treatment in two siCD44 cell lines, suggesting that the AMPK/mTOR pathway was essential for induced autophagy (Fig. 4b). We also performed IHC experiments staining CD44s, caspase-1, p-AMPK, and autophagy marker in HCC specimens, and IHC results were quantified (Fig. 4c, d). Levels of p-AMPK and p62 were increased in HCC specimens characterized by lower expressions of CD44s and caspase-1 (Fig. 4c, d). In conclusion, these findings suggested that activation of the AMPK/mTOR pathway caused by CD44s deficiency was indispensable for autophagy induction and then contributed to the impairment of the caspase-1/IL1B pathway.

### Extracellular IL1B decrease is responsible for impaired EMT phenotype and tumor growth in CD44s deficient HCC cells

**Fig. 5:**
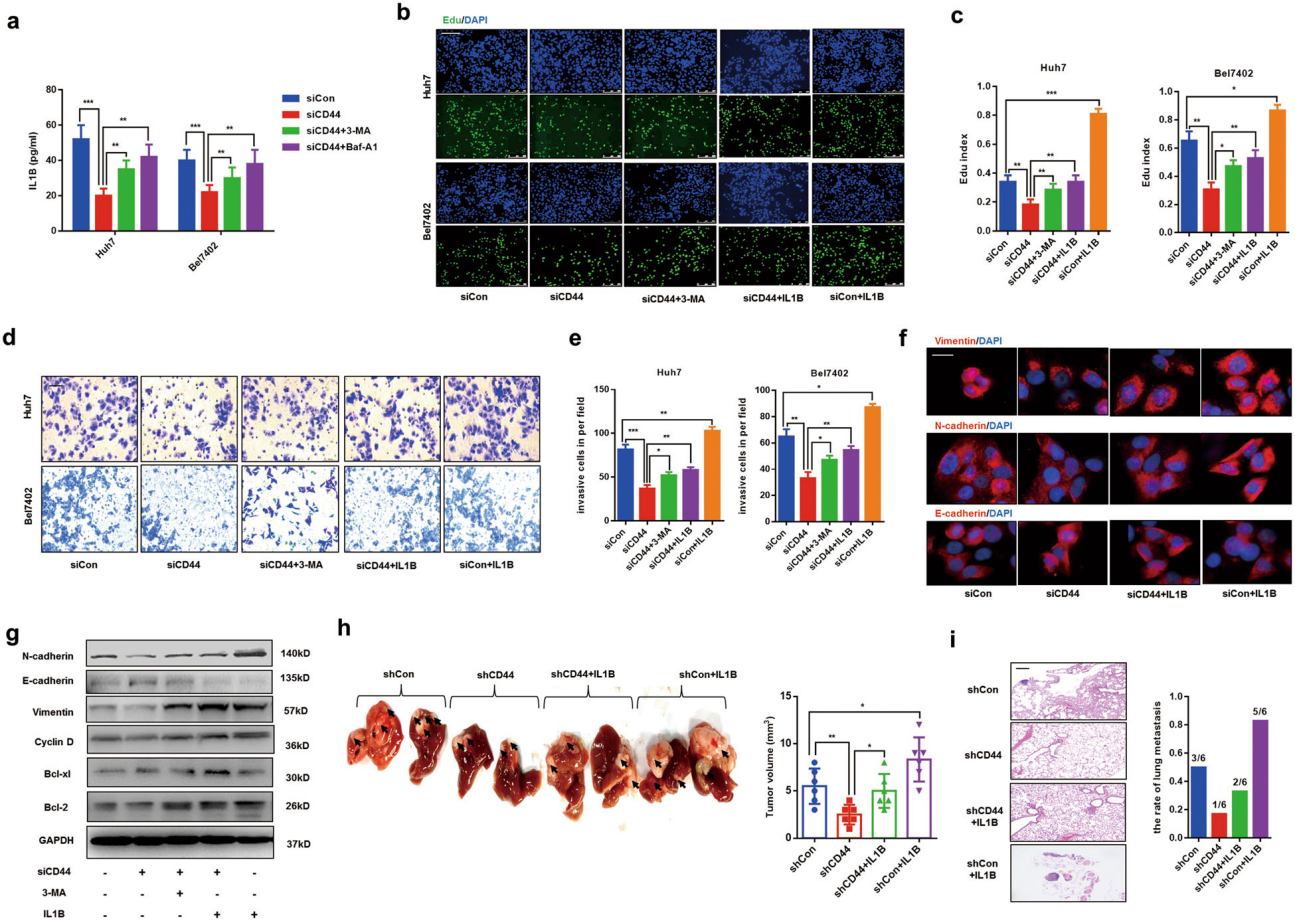
CD44s deficiency-mediated IL1B decrease accounts for EMT inhibition, both in vitro and in vivo. **a** IL1B ELISA analysis was performed, and results indicated that the downregulation of CD44s contributes to the decrease of extracellular IL1B, and autophagy inhibitors such as 3-MA or Baf-A1 rescued the impaired IL1B in siCD44 cells. CD44s deficient cells (siCD44) were treated with 3-MA (10 mM) or Baf-A1 (100 nM) for 24 h. **b**, **c** Edu assays were performed in normal cells (siCon) or CD44s knockdown cells (siCD44), which both were treated with or without human recombinant IL1B for 24 h. Edu index was analyzed in five groups. **d**, **e** Invasion experiments were determined by transwell assays and numbers of invaded cells were counted under the 200× field. **f** EMT-related proteins were analyzed through immunofluorescence staining vimentin, E-cadhrein, and N-cadherin in four groups. **g** Immunoblot analysis indicated that IL1B or 3-MA treatment partly rescued EMT inhibition and proliferation in CD44s deficient cells. **h** HCC orthotopic implantation models indicated IL1B treatment partly recovered tumor growth in nude mice bearing shCD44 cells. The volumes of tumors in corresponding groups were quantified. *n* = 6. **i** Representative images of lung metastasis of three groups were photographed, and the occurrence rate of lung metastasis was counted. *n* = 6. Scale bars, 100 µm. Data are means ± SEM from 3 independent experiments, * means *p* < 0.05, ** means *p* < 0.01, *** means *p* < 0.001 by unpaired student *t* test.

In former work, we had demonstrated the essential role of CD44s in caspase-1/IL1B activation by regulating autophagy. However, in CD44s deficient HCC cells, the role of impaired caspase-1/IL1B in HCC progression remained unclear. Caspase-1 is essential for the maturation and release of IL1B, which plays a critical role in inflammatory diseases^4,23^. Accumulating data demonstrate that IL1B promotes tumor progression and contributes to poor prognosis^28^. In Fig. 5a, we observed that extracellular IL1B was decreased in two siCD44s cells, but both 3-MA and Baf-A1 treatments inhibited the decrease of extracellular IL1B. To explore whether the decrease of released IL1B in CD44s deficient cells was responsible for damaged tumor proliferation and invasion, Bel7402 siCD44 and Huh7 siCD44 cells were treated with human recombinant IL1B for 24 h. Edu assays and transwell experiments were performed, and we found IL1B treatment not only stimulated the abilities of proliferation and invasion in normal cell lines but also rescued the defects of proliferation and invasion in CD44s deficient cells, indicating decreased IL1B was responsible for HCC inhibition caused by CD44s deficiency (Fig. 5b–e).

Emerging evidence has demonstrated that targeting CD44 contributes to defects of metastasis through repressing EMT process^29,30^. Our previous study confirms that HCC inflammatory environment-induced caspase-1 activation is positively associated with EMT phenotype^31^. Given the complicated relationship between CD44, caspase-1, and EMT, we wondered whether IL1B decrease in CD44s deficient cells was responsible for EMT repression. Immunofluorescence staining EMT markers were performed to evaluate EMT phenotype in CD44s deficient cell lines, and results revealed that impaired EMT process was rescued by IL1B treatment (Fig. 5f). Immunoblot analysis was conducted, and we observed that 3-MA or IL1B treatment both recovered damaged protein levels of EMT or proliferation (Fig. 5g). Moreover, Huh7 siCD44 and Bel7402 siCD44 cells were both co-cultured with 3-MA or IL1B for 24 h. As expected, impaired EMT phenotype was partly recovered as well as downregulated expression of caspase-1 (Fig. 5f). To further confirm these findings, experiments of orthotopic implantation of tumors in the liver of nude mice were performed. We observed that, compared to mice bearing tumor tissues collected nude mice injected with Huh7-shCD44 cells subcutaneously, IL1B treatment significantly recovered the damaged tumor growth and metastasis, which were both evidenced by the increasing tumor volumes and rate of lung metastasis (Fig. 5h, i). Moreover, we performed IHC staining CD44s, E-cadherin, Vimentin, and caspase-1 in tumor tissues obtained from the corresponding group of nude mice. We found that IL1B treatment re-expressed caspase-1 and recapitulated EMT phenotype (Fig. S3a, b).

Generally speaking, our findings suggested the decline of released IL1B accounted for impaired EMT phenotype, and growth inhibition, and supplement with IL1B could rescue the defects in HCC progression in CD44s deficient cells.

### Targeting CD44s abrogates hypoxia-induced caspase-1/IL1B through decreasing HIF1A and enhancing autophagic activity

**Fig. 6:**
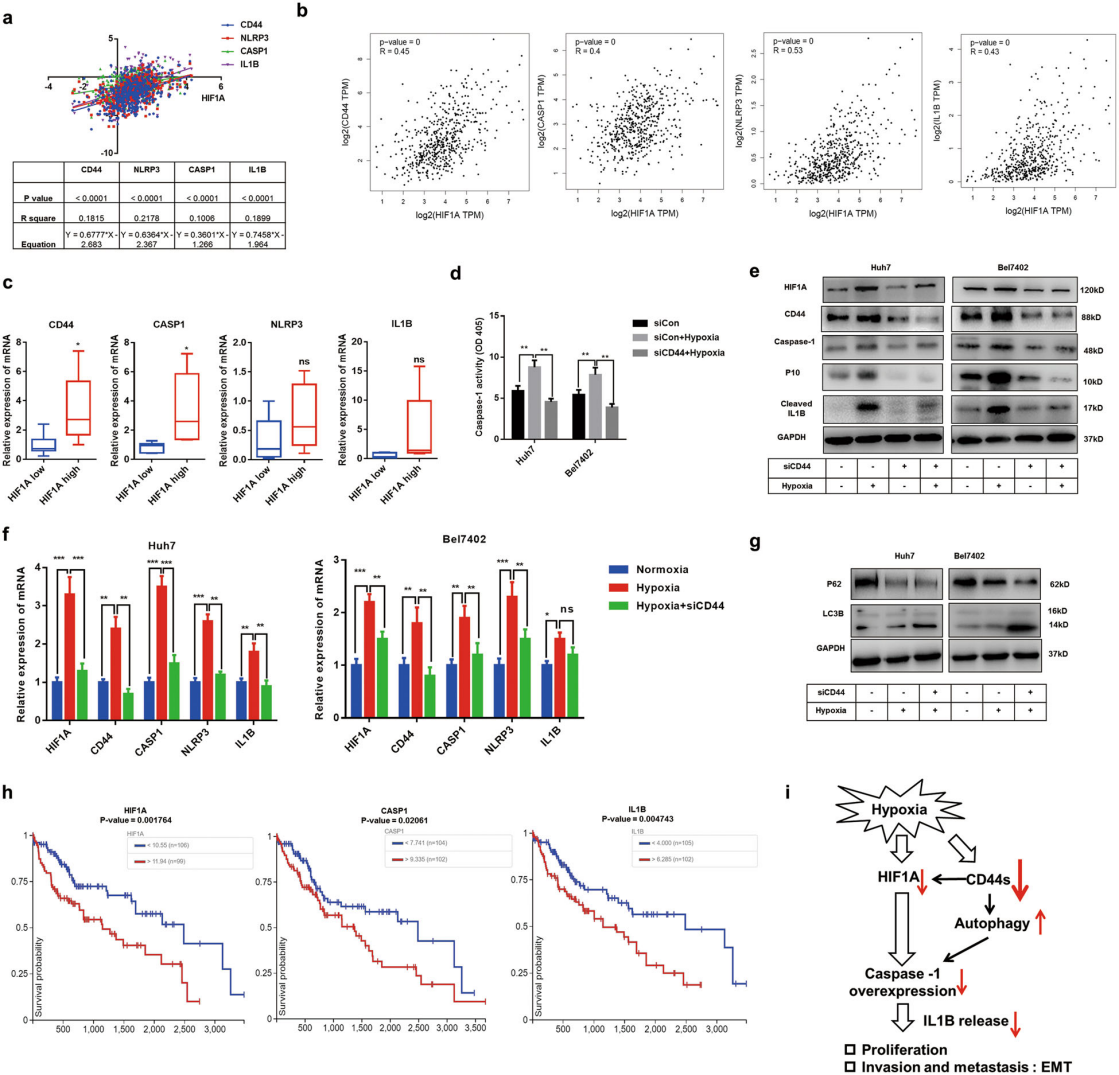
Targeting CD44s represses hypoxia-induced caspase-1/IL1B via decreasing HIF1A and increasing autophagy. **a** A positive correlation between HIF1A, CD44, and genes involved in the caspase-1/IL1B pathway. Relevant data were extracted from UCSC Xena for correlation analysis. **b** A positive correlation between HIF1A, CD44, and genes involved in the caspase-1/IL1B pathway. Relevant data were extracted from GEPIA for correlation analysis. **c** mRNA expression of CD44 and genes involved in the caspase-1/IL1B pathway in HCC tissues with high or low HIF1A expression. *n* = 50. **d** Caspase-1 activity analyses were performed in three groups, and results suggested that siCD44 treatment inhibited hypoxia-induced caspase-1 capacity. **e**, **f** Q-PCR and immunoblot analysis both indicated the downregulation of CD44s inhibits hypoxia-induced caspase-1 expression via reducing HIF1A. **g** Immunoblot analysis suggested targeting CD44s further promoted autophagy under hypoxia condition. **h** HIF1A, CASP1, and IL1B expression are significantly related to OS in HCC. Data were extracted from the UCSC Xena database. **i** The schematic outline of this study. In normoxia conditions, CD44s deficiency promotes p62-mediated autophagic degradation of caspase-1, which results in suppression of the caspase-1/IL1B pathway. In hypoxia conditions, targeting CD44s inhibits hypoxia-induced activation of caspase-1/IL1B through decreasing HIF1A and increasing autophagy. Data are means ± SEM from 3 independent experiments, * means *p* < 0.05, ** means *p* < 0.01, *** means *p* < 0.001 by unpaired student *t* test.

Hypoxia has been demonstrated to induce caspase-1 activation and IL1B release^5,7^. In addition, hypoxia could promote CSCs formation by upregulating CD44 in varied tumors^32–34^. Hypoxia-induced factor 1 alpha (HIF1A) is a crucial protein controlling hypoxia response. We had demonstrated CD44s modulated caspase-1/IL1B via enhancing autophagy level in normoxia conditions. However, the role of CD44s in hypoxia-induced caspase-1/IL1B is not clear. To determine the mRNA relationship between HIF1A, CD44, and genes involved in caspase-1/IL1B, public data from UCSC Xena or GEPIA were collected and analyzed, results of which indicated that HIF1A was positively associated with CD44 and genes involved in the caspase-1/IL1B pathway (Fig. 6a, b). Moreover, by analyzing related mRNA expressions in 50 HCC tissues, we confirmed that HIF1A was positively correlated to CD44 and CASP1 instead of NLRP3 or IL1B (Fig. 6c). In accordance with previous studies, we confirmed that hypoxia upregulated CD44s activated the caspase-1/IL1B pathway and enhanced caspase-1 capacity in both two HCC cells lines (Fig. 6d–f). To further demonstrate the relationship of CD44s and caspase-1/IL1B in hypoxia condition, Huh7 siCD44, and Bel7402 siCD44 cells were cultured under hypoxia for 24 h. In contrast to normal HCC cells, we observed that CD44s deficiency significantly suppressed hypoxia-induced caspase-1/IL1B activation and caspase-1 activity, suggesting that CD44s regulated caspase-1/IL1B pathway both in normoxia and hypoxia conditions (Fig. 6d–f). Interestingly, HIF1A was inhibited by CD44 deficiency in hypoxia conditions both in mRNA and protein levels (Fig. 6e, f). These findings revealed that targeting CD44s inhibited hypoxia-induced caspase-1/IL1B by decreasing HIF1A.

In former work, we had demonstrated the essential role of autophagy in caspase-1/IL1B repression caused by CD44s deficiency in normoxia conditions. To explore the role of autophagy in hypoxia-induced caspase-1/IL1B activation, immunoblot and Q-PCR analyses were performed, and we observed that autophagy levels in two HCC cell lines were enhanced under hypoxia conditions and targeting CD44s could further increase autophagic activities, suggesting that CD44s deficiency contributed to autophagy induction and IL1B decrease both in normoxia and hypoxia conditions (Fig. 6g, Fig. S4a, b). Lastly, to determine the predictive role of HIF1A and caspase-1/IL1B, data from TCGA public databases were extracted and evaluated. We found that HCC patients with high expression of HIF1A, caspase-1, and IL1B were characterized by poor prognosis (Fig. 6h).

Our study indicated that, on the one hand, CD44s were positively associated with caspase-1/IL1B pathway, and targeting CD44s promoted p62-mediated degradation of caspase-1 through activating AMPK/mTOR signaling and then decreased IL1B release in normoxia conditions, which contributed to the impairment of EMT phenotype and tumor proliferation. On the other hand, we confirmed that CD44s deficiency abolished hypoxia-induced caspase-1/IL1B activation via decreasing HIF1A and enhancing autophagy. Taken together, we identify a novel regulatory mechanism between CD44s and caspase-1/IL1B pathway, which provides potential therapeutic targets for HCC inhibition (Fig. 6i).

## Discussion

Mounting evidence has indicated that caspase-1/IL1B signaling is involved in HCC proliferation, invasion, and inflammatory environment^4–6^. In our study, we observed that caspase-1, instead of NLRP3 or IL1B, was positively associated with CD44s both in HCC specimens and cell lines. Moreover, CD44s knockdown significantly inhibited caspase-1 expression, caspase-1 activity, and IL1B maturation, suggesting the the caspase-1/IL1B pathway was inactivated.

CD44s is overexpressed in HCC tissues and correlated with poor prognosis^35–37^. We observed that autophagy levels were increased in two CD44s deficient cells. Autophagy has been reported to be involved in regulating the caspase-1/IL1B pathway. Dupont et al.^38^ reported that an autophagy-based secretory pathway is essential for IL1B release in macrophage. With the treatment of 3-MA or CQ, damaged the caspasse-1/IL1B pathway and decreased caspase-1 activity were both rescued, which suggested that autophagy was responsible for impairment in caspase-1/IL1B mediated by CD44s deficiency.

Autophagy is considered as a conserved degradation system in cells^39^. Autophagy-mediated NLRP3 degradation was found in macrophage treated by dopamine, which inhibits systemic inflammation^22^. In addition, accumulating data indicate the correlation between CD44 and NLRP3 activation in inflammatory diseases^40,41^, which indicated targeting CD44 inhibits NLRP3–caspase-1-IL1B signaling by reducing NF-κB p65 subunit nuclear translocation or inhibition of reactive oxygen species generation. However, we found caspase-1 instead of NLRP3 or IL1B has directly interacted to p62 in HCC cells. Moreover, CD44s deficiency strengthened the interaction between p62 and caspase-1, which resulted in the degradation of caspase-1 and then inhibited the caspase-1/IL1B pathway. Therefore, our findings revealed that p62-mediated autophagic selective degradation of caspase-1 was responsible for the inactivation of caspase-1/IL1B and decreased caspase-1 activity.

Several studies indicate AMPK signaling participates in the formation of CD44-positive CSC, and targeting CD44 could result in tumor inhibition by inducing AMPK activation^42–44^. Moreover, AMPK/mTOR signaling has been determined to be crucial upstream regulators of autophagy^45^. We explored the relationship between CD44s and AMPK and found that AMPK/mTOR signaling was activated in CD44s deficient HCC cells. With the treatment of AMPK siRNA, autophagy induced by CD44s deficiency was repressed, and impaired caspase-1/IL1B was rescued, which indicated that the AMPK/mTOR pathway was essential for suppression of the caspase-1/IL1B pathway by increasing autophagy.

In HCC, CD44s are responsible for the acquisition of the mesenchymal phenotype and maintenance of CSCs^10,11,30,46^. We demonstrated that CD44s deficiency-induced autophagy induction was essential for impaired EMT phenotype, invasion and proliferation. With the treatment of 3-MA, the defects in EMT phenotype, invasion, and proliferation caused by CD44s deficiency were recovered both in vivo and in vitro. Furthermore, accumulating data demonstrate the promoting role of IL1B in tumor development. A recent study reported that IL1B could promote proliferation and EMT phenotype in colorectal carcinoma^28^. With the treatment of recombination human IL1B, we observed impairment of EMT phenotype, invasion, and proliferation in CD44s deficient cells were rescued, which suggested that CD44s deficiency-mediated IL1B decrease contributed to the inhibition of HCC progression.

HIF1A is a crucial regulator in increasing autophagy and CSCs^47,48^. In hypoxic breast cancers, CD44s overexpression facilitates tumor proliferation and invasion by enhancing HIF1A signaling^32^. Hypoxia-induced caspase-1 activation has been reported to promote HCC progression in an extracellular HMGB1 dependent way^5^. Here, we confirmed hypoxia increased CD44s, autophagy, and activated the caspase-1/IL1B pathway. We further demonstrated that caspasse-1/IL1B could be inhibited by targeting CD44s through downregulating HIF1A and enhancing autophagy in hypoxia conditions.

In conclusion, we demonstrate there is a positive relationship between CD44s and the caspase-1/IL1B pathway in HCC. CD44s knockdown inhibits HCC proliferation and EMT phenotype by increasing autophagy. We further confirm that p62-mediated autophagic degradation of caspase-1 is responsible for caspase-1/IL1B suppression in CD44s deficient cells. Caspase-1/IL1B pathway and caspase-1 activity could be regulated by CD44 both in normoxia and hypoxia conditions. Taken together, our study elucidates the novel regulatory molecular mechanism of caspase-1/IL1B by CD44s, and these findings might provide potential therapeutic targets for HCC inhibition.

## Supplementary information

Figure S1. CD44s correlates with caspase-1 expression in HCC tissues

Figure S2. Targeting CD44s leads to autophagy induction

Figure S3. IL1B recovers impaired EMT phenotype caused by CD44s deficency

Figure S4. Targeting CD44s further strengthened autophagic activity in hypoxia conditions

Figure S5. Negative controls of IHC and IF experiments

Supplementary figure legends and table

## Data Availability

The data supporting the findings of this study are available within the article and supplementary files or from the authors upon reasonable request.
