# Supplementary figures and images for: The novel interplay between CD44 standard isoform and the caspase-1/IL1B pathway to induce hepatocellular carcinoma progression
Source: Cell Death Dis. 2020 Nov 9;11(11):961. doi: 10.1038/s41419-020-03158-6 (PMC7652828; doi:10.1038/s41419-020-03158-6)

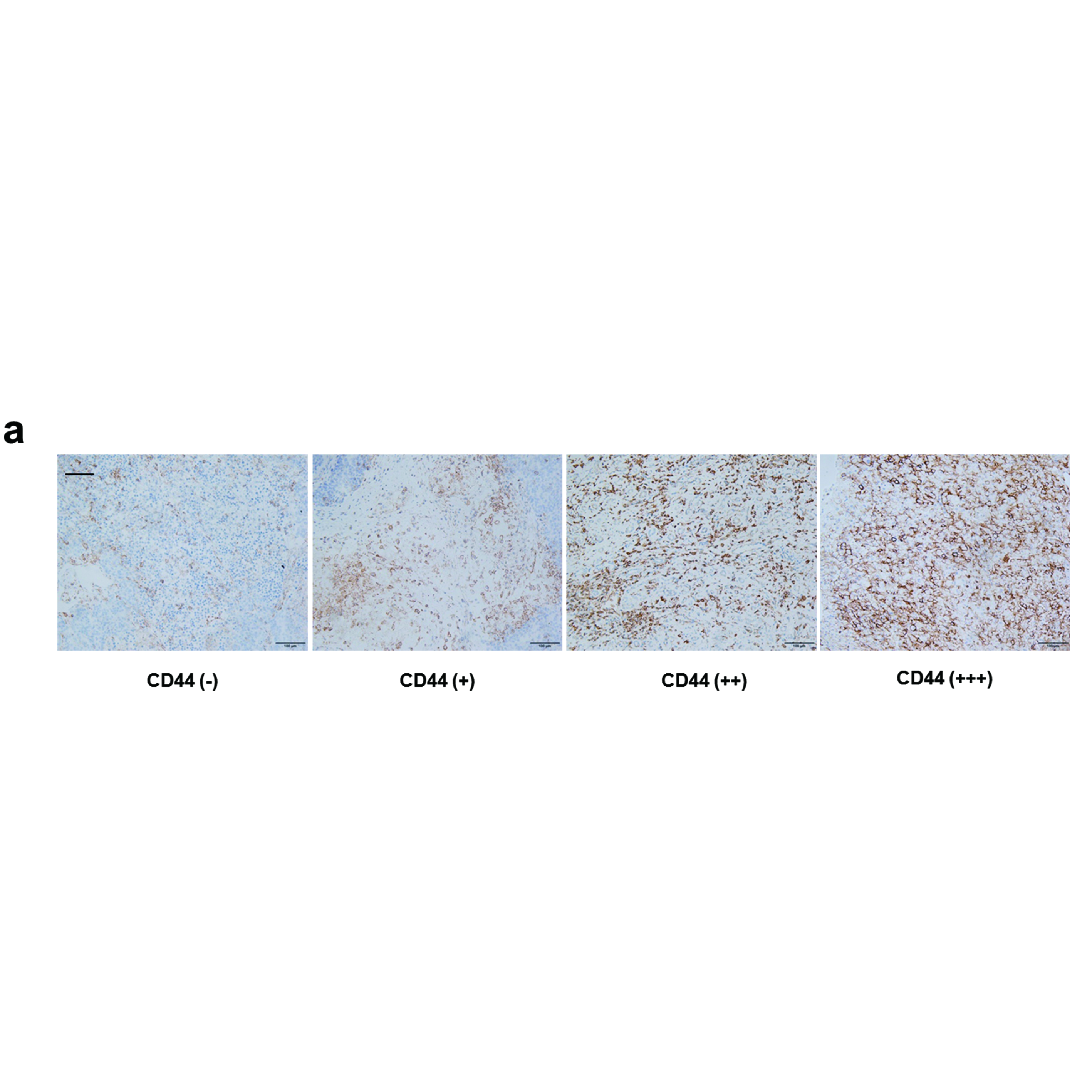

Supplement: Supplementary file 1 — Figure S1. CD44s correlates with caspase-1 expression in HCC tissues [file 41419_2020_3158_MOESM1_ESM.tif]

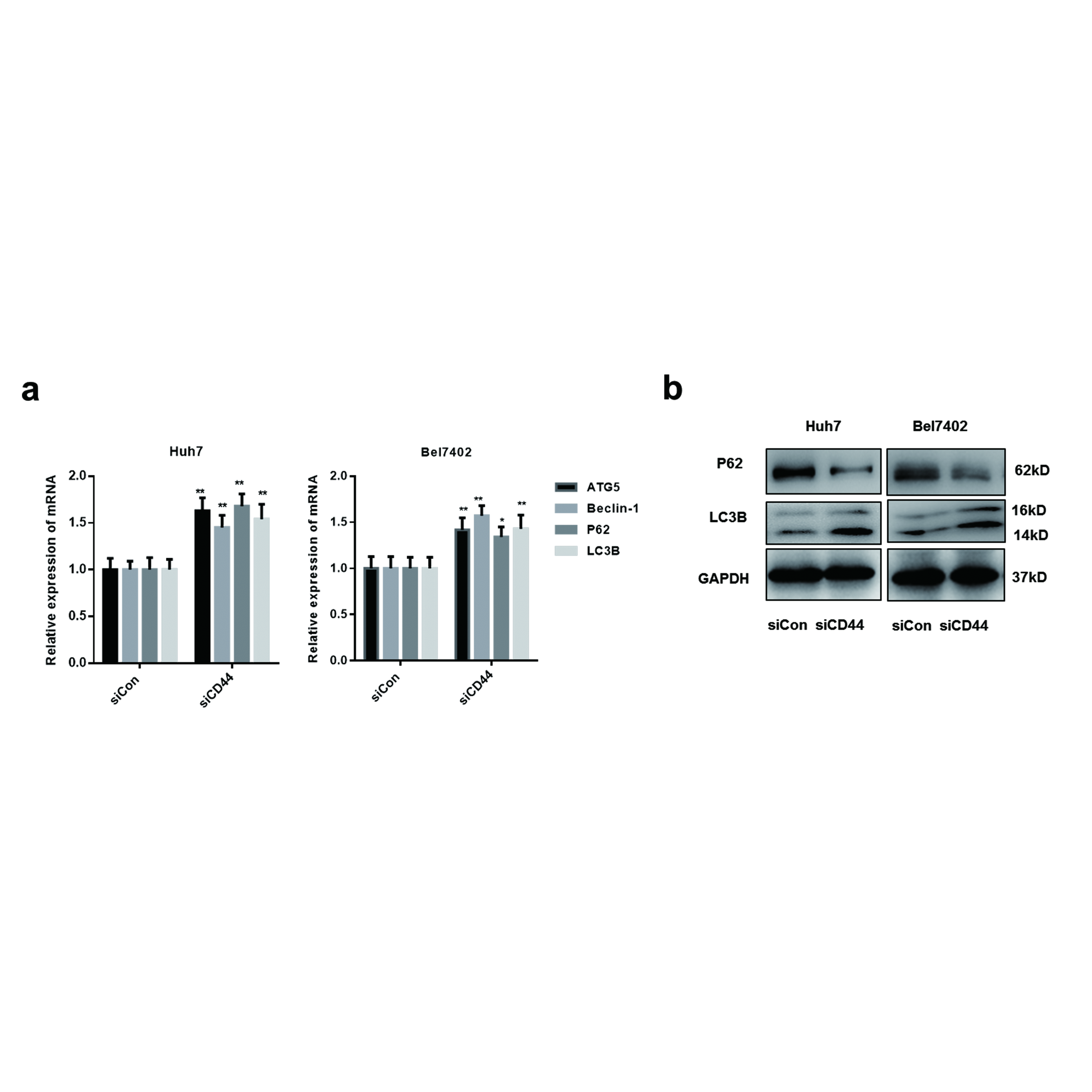

Supplement: Supplementary file 2 — Figure S2. Targeting CD44s leads to autophagy induction [file 41419_2020_3158_MOESM2_ESM.tif]

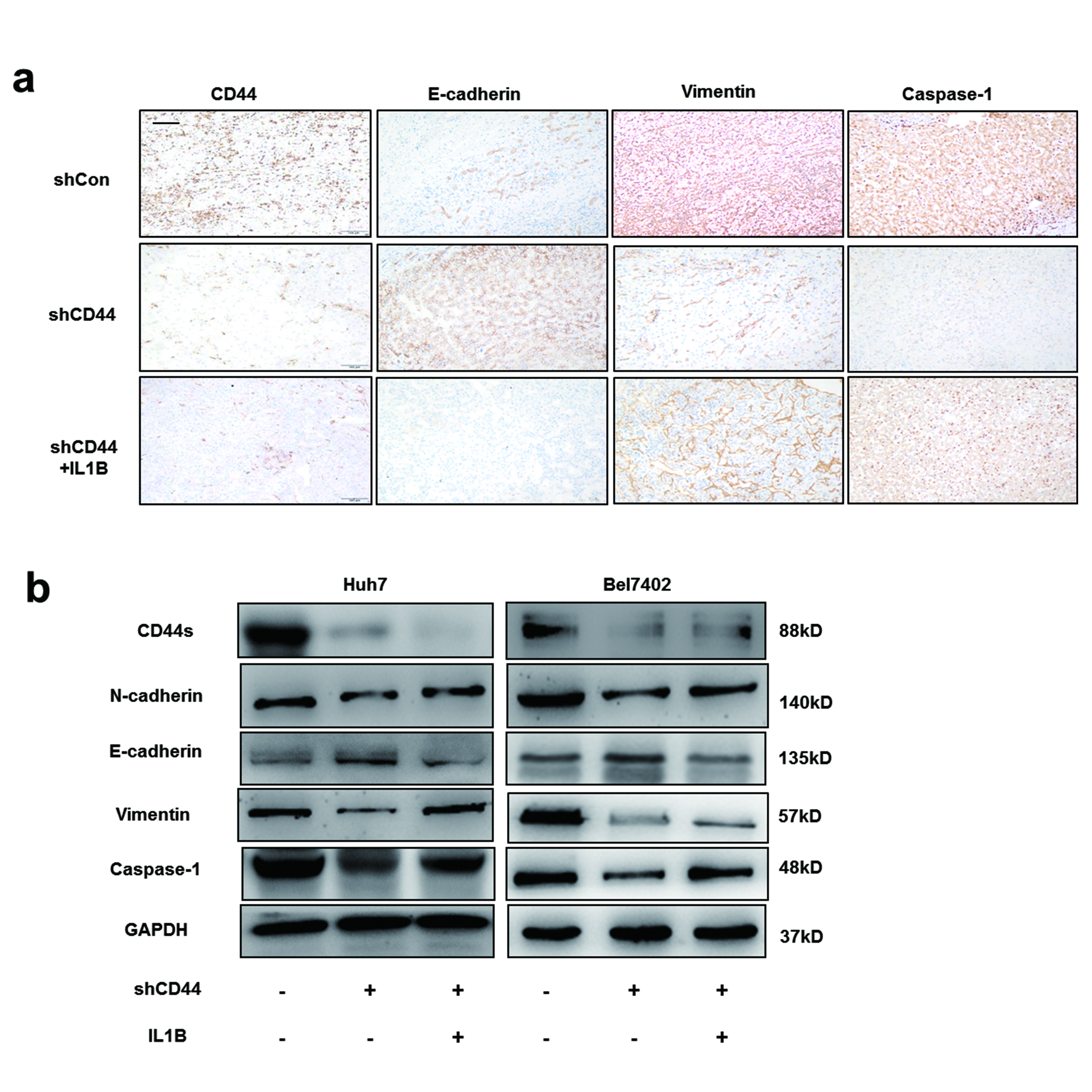

Supplement: Supplementary file 3 — Figure S3. IL1B recovers impaired EMT phenotype caused by CD44s deficency [file 41419_2020_3158_MOESM3_ESM.tif]

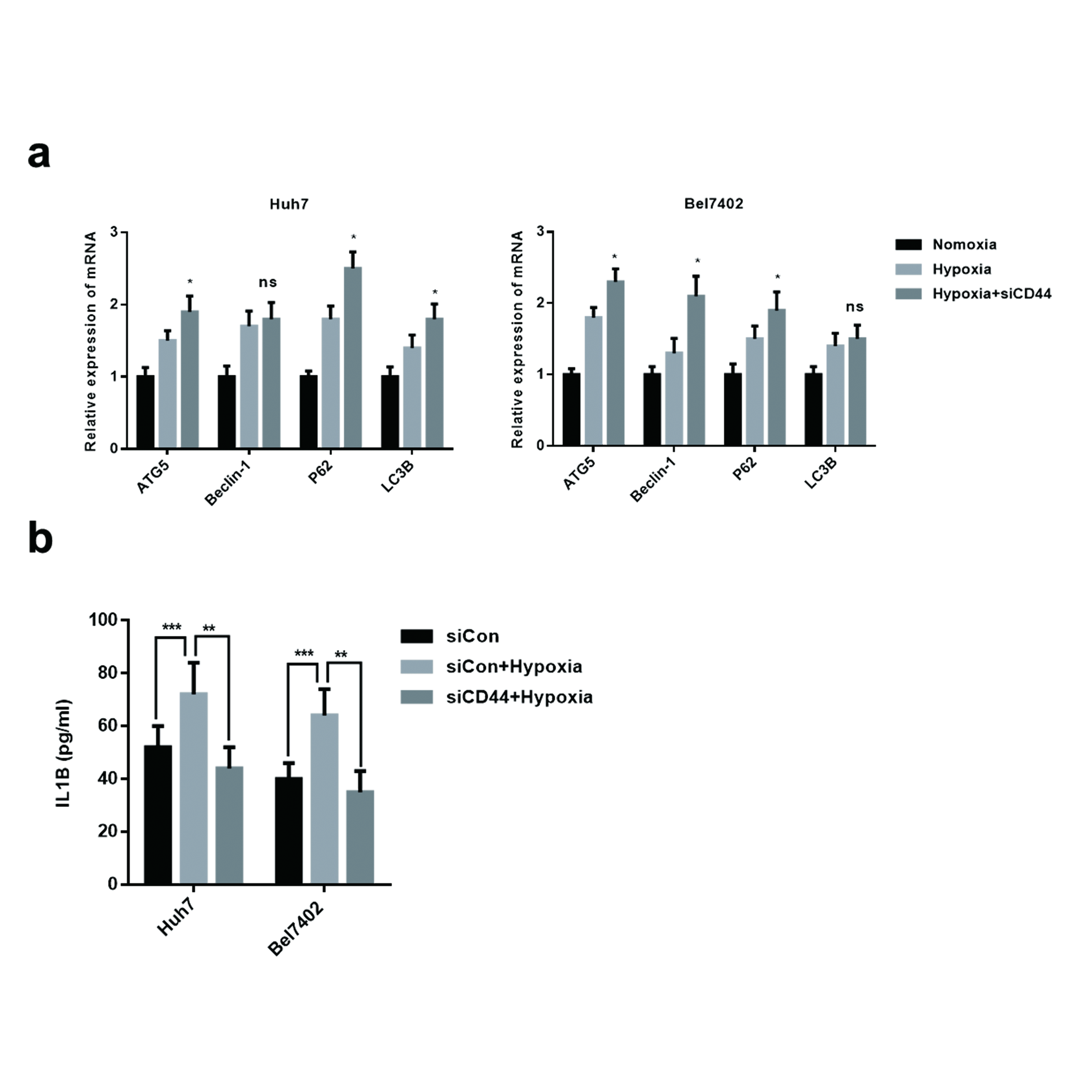

Supplement: Supplementary file 4 — Figure S4. Targeting CD44s further strengthened autophagic activity in hypoxia conditions [file 41419_2020_3158_MOESM4_ESM.tif]

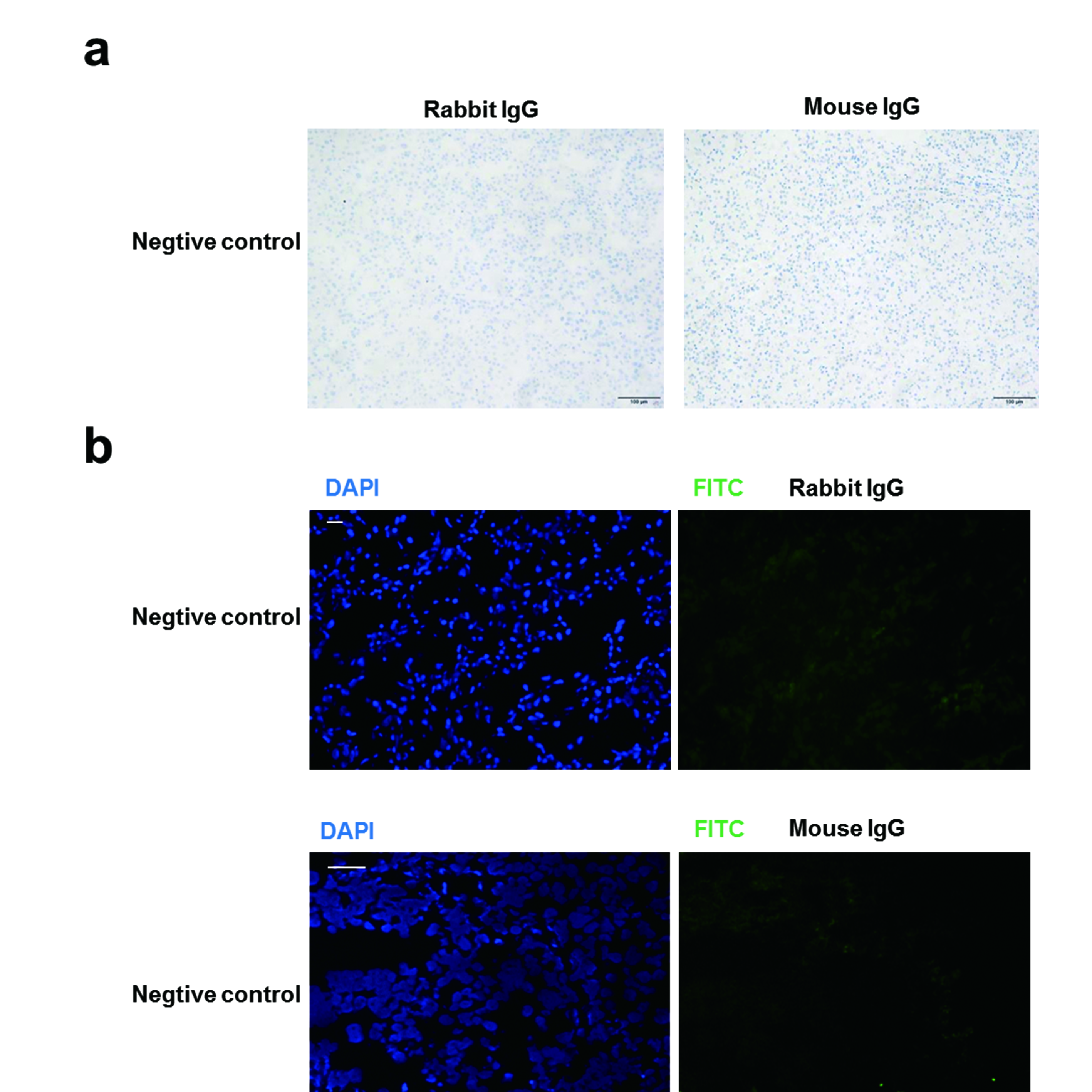

Supplement: Supplementary file 5 — Figure S5. Negative controls of IHC and IF experiments [file 41419_2020_3158_MOESM5_ESM.tif]
