# Supplementary material for: The novel interplay between CD44 standard isoform and the caspase-1/IL1B pathway to induce hepatocellular carcinoma progression
Source: Cell Death Dis. 2020 Nov 9;11(11):961. doi: 10.1038/s41419-020-03158-6 (PMC7652828; doi:10.1038/s41419-020-03158-6)
Supplement: Supplementary file 6 — Supplementary figure legends and table [file 41419_2020_3158_MOESM6_ESM.docx]

**SUPPLEMENTARY MATERIALS**

**Supplementary Table 1. Antibodies and reagents**

**Supplementary Table 2. Q-PCR primer sequences**

| Gene | Forward ­­ | Reverse |
| --- | --- | --- |
| 18s | CGGCG ACGACCCATTCGAAC | GAATCGAACCCTGATTCCCCGTC |
| HIF1A | ATCCATGTGACCATGAGGAAATG | TCGGCTAGTTAGGGTACACTTC |
| CD44 | GCAGTCAACAGTCGAAGAAGG | TGTCCTCCACAGCTCCATT |
| CASP1 | TTTCCGCAAGGTTCGATTTTCA | GGCATCTGCGCTCTACCATC |
| NLRP3 | GATCTTCGCTGCGATCAACAG | CGTGCATTATCTGAACCCCAC |
| IL1B | TTCGACACATGGGATAACGAGG | TTTTTGCTGTGAGTCCCGGAG |
| Lc3B | AGCAGCATCCAACCAAAATC | CTGTGTCCGTTCACCAACAG |
| P62 | ATCGGAGGATCCGAGTGT | TGGCTGTGAGCTGCTCTT |
| Beclin-1 | CAGGAGAGACCCAGGAGGAA | GCTGTTGGCACTTTCTGTGG |
| ATG5 | AGAAGCTGTTTCGTCCTGTGG | AGGTGTTTCCAACATTGGCTC |

**Supplementary Figure Legends**

Fig.S1. CD44s correlates with caspase-1 expression in HCC tissues

(a) Representative images of defferent intensities IHC staining CD44s.

Fig.S2. Targeting CD44s leads to autophagy induction

(a-b) Q-PCR and immunoblot analysis were both performed and results suggested CD44s deficiency promoted autophagy. Data are means ± SEM from 3 independent experiments, * means p<0.05, ** means p<0.01, *** means p<0.001 by unpaired student T test.

Fig.S3. (a) Representative images showed different intensities of CD44s, caspase-1, E-cadhern and Vimentin in tumor tissues collected from three groups in nude mice. Scale bars, 100um. (b) Immunoblot analysis were both performed and results suggested IL1B treatment recovered damaged EMT phenotype caused by CD44s deficiency.

Fig.S4. Targeting CD44s further strengthened autophagic activity in hypoxia conditions.

(a) Q-PCR analysis was performed and results indicated CD44s deficiency enhanced autophagic activity in hypoxia conditions. (b) IL1B elisa analysis was performed and results indicated that CD44s deficiency repressed hypoxia-induced IL1B release. Data are means ± SEM from 3 independent experiments, * means p<0.05, ** means p<0.01, *** means p<0.001 by unpaired student T test.

Fig.S5. (a-b) Negative controls of IHC and IF experiments.
